# Dropping Out or Keeping Up? Early-Dropouts, Late-Dropouts, and Maintainers Differ in Their Automatic Evaluations of Exercise Already before a 14-Week Exercise Course

**DOI:** 10.3389/fpsyg.2016.00838

**Published:** 2016-06-02

**Authors:** Franziska Antoniewicz, Ralf Brand

**Affiliations:** Division of Sport and Exercise Psychology, University of PotsdamPotsdam, Germany

**Keywords:** exercise adherence, automatic evaluations, BIAT, dropout, associations, affect

## Abstract

The aim of this study was to examine how automatic evaluations of exercising (AEE) varied according to adherence to an exercise program. Eighty-eight participants (24.98 years ± 6.88; 51.1% female) completed a Brief-Implicit Association Task assessing their AEE, positive and negative associations to exercising at the beginning of a 3-month exercise program. Attendance data were collected for all participants and used in a cluster analysis of adherence patterns. Three different adherence patterns (52 maintainers, 16 early dropouts, 20 late dropouts; 40.91% overall dropouts) were detected using cluster analyses. Participants from these three clusters differed significantly with regard to their positive and negative associations to exercising before the first course meeting ($\eta_{p}^{2}$ = 0.07). Discriminant function analyses revealed that positive associations to exercising was a particularly good discriminating factor. This is the first study to provide evidence of the differential impact of positive and negative associations on exercise behavior over the medium term. The findings contribute to theoretical understanding of evaluative processes from a dual-process perspective and may provide a basis for targeted interventions.

## Introduction

Automatic evaluations of exercising (AEE; i.e., the spontaneous associations of exercising with either positive or negative affect) are a fairly well-researched phenomenon (e.g., Bluemke et al., 2010; Antoniewicz and Brand, 2014). Engagement in exercise is not just a consequence of deliberate reasoning but also the result of unintentional, automatic evaluations. Most empirical research on AEE has focused on correlations between AEE and exercise volumes (Bluemke et al., 2010; Conroy et al., 2010), the predictive power of AEE in relation to proximal episodes of physical activity (e.g., step counts for 1 week, Conroy et al., 2010) or decisions about exercising (Antoniewicz and Brand, 2014; Brand and Schweizer, 2015). Other research has investigated changes in AEE (Markland et al., 2015; Antoniewicz and Brand, 2016); however, the potential role of AEE in exercise maintenance has not been researched before.

Non-adherence to exercise programs is a well documented phenomenon (e.g., Marcus et al., 2000). Dropout rates of approximately 50% after a couple months are not uncommon (Matsumoto and Takenaka, 2004). We think that research on the psychological variables that influence the behavioral decisions of maintainers and non-maintainers is crucial to designing and implementing successful exercise interventions.

This study aimed to address a significant research gap by (1) providing a theoretical account of the role of AEE in exercise adherence and (2) testing a hypothesis relating exercise adherence to AEE which was derived from this account.

### The Role of Automatic Evaluations of Exercising in Exercise Maintenance

According to dual process models of social cognition evaluative reactions involve two interconnected evaluative processes: one spontaneous and automatic, the other rather thoughtful and deliberative (Chaiken and Trope, 1999; Kahneman and Frederick, 2002). The Reflective Impulsive Model (RIM; Strack and Deutsch, 2004) represents one attempt to explain the distinction. According to this model, an individual who comes to the conclusion that the advantages of exercising today (e.g., thinking about health benefits) outweigh the disadvantages (e.g., exercise is time-consuming) will develop an intention to exercise that evening. The RIM assigns this process to the reflective system. The model further assumes that at the same time as the reflective processing AEE (e.g., doing aerobics is enjoyable) arise as a result of activation of an associative network which is part of the impulsive system. These AEE elicit a corresponding motivational orientation (e.g., I want to attend an aerobic session today). This aspect of social cognition – the role of the impulsive system – is central to our study.

Automatic evaluations of exercising represent learnt associations, which serve as a “conceptual and procedural long-term memory, where associative weights between contents change slowly and gradually” (Deutsch and Strack, 2006, p. 167) and according to RIM the salience and accessibility of these associative clusters varies according to the frequency of their activation. One would expect a regular exerciser to have strong, easily accessible positive affective associations to exercising and weaker negative affective associations to exercising.

Exercising is massively associated with bodily sensations and evokes affective responses (Ekkekakis et al., 2011). Affective states provide information and can be registered in memory (Clore, 1992; Ekkekakis et al., 2016). External stimuli (e.g., the experience of entering the gym) and internal stimuli (e.g., thinking about exercising) can activate affective representations which serve as inputs to evaluative information processing. Findings from exercise psychology show that positive affect during and after exercise predicts future exercising (e.g., Ekkekakis et al., 2011), and that positive AEE, as well as unconnected evaluative judgments, influence immediate decisions about exercising (Brand and Schweizer, 2015). Repeated activation of stored affective representations by acute affective states, experienced during and shortly after exercise, reinforces their association with exercise (Deutsch and Strack, 2006), i.e., increases the strength of the association between mental representations of exercise behavior and affective evaluative concepts. Every time an individual has to decide whether or not to attend the exercise course that day, both reflective and impulsive evaluative processes contribute to the formation of a motivational orientation (in the impulsive system) and a behavioral intention (in the reflective system), and reinforce pre-existing affective representations associated with attending the exercise course. This learning cycle is the reason why we expected to find predominantly positive spontaneous AEE in persistent exercisers.

### Researching Automatic Evaluations of Exercising

Individuals are often unaware of their automatic associations (Nosek et al., 2007) and data based on questionnaires that ask participants to introspect about such associations are therefore not an appropriate or valid measure of them, so over the past 20 years researchers have begun to investigate the validity of response time latency tasks (Fazio and Olson, 2003) as indicators of automatic associations. These indirect tests, e.g., the Affective Priming Task (Fazio et al., 1995) and the Implicit Association Test (IAT; Greenwald et al., 1998), infer the individuals’ AEE from the speed with which they categorize word or picture stimuli into various categories.

#### The Implicit Association Test

Over the past two decades the IAT (Greenwald et al., 1998) has become recognized in social psychology as a standard measure of spontaneous associations between mental concepts (it should be noted, however, that there is active debate on the automaticity of the measured reactions; De Houwer et al., 2009). The standard version of the IAT uses sets of stimuli related to two complementary targets (e.g., ‘exercise’ vs. ‘non-exercise’) or two complementary evaluative categories (‘good’ vs. ‘bad’). The respondent has to sort the stimuli as quickly and accurately as possible into combined categories which are varied systematically across blocks (e.g., stimuli representing ‘exercise’ or ‘good’ are moved to the left side whilst stimuli representing ‘non-exercise’ or ‘bad’ stimuli are moved to the right side in test block A; whereas in test block B ‘exercise’ and ‘bad’ stimuli have to be sorted to the left side and ‘non-exercise’ and ‘good’ stimuli are sorted to the right side). Research from exercise psychology indicates that there is no clear conceptual opposite of ‘physical activity’ (e.g., Rebar et al., 2015). The brief IAT (BIAT; Sriram and Greenwald, 2009) is a version of the IAT which addresses this issue. In the BIAT participants only have to pay attention to two out of four categories in each test block (i.e., detect whether stimuli represent the concepts of, e.g., ‘exercise’ or ‘good’ in one test block and ‘exercise’ or ‘bad’ in the other). This makes features of the non-focal category (‘non-exercise’) less important. Another approach to addressing the lack of a complement to the target category is the Single Category (SC)-IAT (Karpinski and Steinman, 2006). In one SC-IAT block respondents decide whether stimuli belong to the categories ‘exercise’ or ‘good’ *or* to the category ‘bad’; in the other test block they decide whether stimuli belong to the ‘exercise’ or ‘bad’ categories *or* to the evaluative category ‘good.’

The common assumption underlying all IATs is that when stimuli sharing the same response are compatible (e.g., for participants who evaluate exercising positively the same response is required to stimuli representing ‘exercise’ or ‘good’) stimuli are handled more quickly than when the categorization is incompatible with one’s automatic evaluation. Test scores are usually calculated by subtracting mean reaction times from the incompatible block from those in the compatible block – the two associative foci (Sriram and Greenwald, 2009) – divided by the pooled standard deviation from blocks. The resulting relative difference score (*D*-score; Greenwald et al., 2003) is an effect size-like measure. Thinking of a continuum between either having spontaneous negative affective evaluations with exercising or positive affective evaluations, the *D*-scores resembles one score along this continuum. A positive *D*-score can be interpreted as relatively positive AEE.

#### Selected Relevant Studies

A few exercise psychology studies have already used IATs and the *D*-score to illustrate the relationships between automatic evaluations from various forms of physical activity (e.g., Conroy et al., 2010; Hyde et al., 2012; Antoniewicz and Brand, 2016).

Conroy et al. (2010) employed the SC-IAT to show that more positive *D*-scores (i.e., faster reactions to ‘good’ stimuli when the same response is required for stimuli belonging to the target category of exercise) were associated with higher physical activity (number of steps per day) after controlling for well-established predictors of physical activity (e.g., self-efficacy). The authors concluded that spontaneous physical activity behavior over a short timeframe - 1 week - was influenced by both reflective motivational processes and impulsive processes.

Hyde et al. (2012) used the same length of observation period, 1 week, and focused on the stability of participants’ automatic evaluations. At the beginning and end of the 1-week period participants worked through a SC-IAT and reported their physical activity during the previous week. Changes in *D*-score indicating a shift to a more favorable AEE were accompanied by an increase in physical activity level. The authors concluded that AEE include stable and more temporally variable components, both of which are related to physical activity behavior.

Antoniewicz and Brand (2016) investigated variability in AEE by manipulating participants’ AEE with an Evaluative Conditioning Task. They assessed changes in SC-IAT *D*-scores immediately after the manipulation in three experimental groups (associating exercise with positive affect; associating exercise with negative affect; control condition). The manipulation was shown to be effective; *D*-scores were significantly higher in the group that learned positive AEE than in the control group. Drawing on theories positing that the impulsive system is based on associative processes (Deutsch and Strack, 2006) and the proposed interpretation of *D*-scores (Greenwald et al., 2003) the authors distinguished between the two *D*-score components (i.e., associations between ‘exercise-good’ – the positive associative focus and ‘exercise-bad’ – the negative associative focus) and analyzed their manipulation induced changes. This revealed that the observed learning was mainly driven by changes during the ‘exercise-bad’ association rather than the ‘exercise-good’ association. The authors interpreted this as an indication that amongst their sports student participants the ‘exercise-good’ association was relatively stable, whilst the ‘exercise-bad’ association was more susceptible to manipulation.

### This Study

This study aimed to address a significant research gap by investigating the influence of AEE on adherence to a long-term exercise program. We monitored participants’ adherence to a 3-month program of exercise (classifying them according to adherence, e.g., dropouts vs. maintainers) and assessed their baseline spontaneous evaluative associations with exercise.

According to dual process theories such as the RIM, the motivational orientation toward exercise (e.g., approach or avoid exercise) is based on the difference between the weights of ‘exercise-good’ associations and ‘exercise-bad’ associations. There is no doubt that exercising can simultaneously have both positive and negative associations for an individual. Regular participation in an aerobics class might elicit positive affect when it evokes thoughts of the friends one meets there whilst also eliciting more negative affect related to the resulting muscle ache. It is our contention that although a relative measure such as BIAT *D*-score might reflect AEE and hence capture differences between exercisers and non-exercisers as shown before, the initially measured *D*-score might be too robust to reflect the more nuanced differences between people who start an exercise program and adhere (i.e., maintainers) and people who do the same and quit in the long run (i.e., dropouts). We assume that it could be useful to conceive AEE in terms of separate exercise-positive and exercise-negative associations rather than using combined measures (e.g., *D*-score).

Carrying forward findings from previous studies (Antoniewicz and Brand, 2016) we expected to find inter-individual differences not only between exercisers and non-exercisers but also between finer behavioral sub-groups (e.g., sporadic vs. frequent exercisers) on the level of the two more nuanced exercise-positive and exercise-negative associations. We hypothesized that at baseline (before the start of the exercise program) positive associations (i.e., a positive associative focus) would be stronger in participants who would subsequently persist with the program than in those who would drop out.

## Materials and Methods

### Participants

#### Sample

Data were collected from 121 participants. Data from some participants were excluded from analysis because of problems understanding the instructions for the tests (*n* = 20), because participants had left the exercise program ahead of schedule for health reasons (*n* = 2), because they had an error rate of more than 20% in BIAT sorting trials (*n* = 7) or because they reported before the program that they had little intention of finishing the program (*n* = 4). Intention to finish the program was assessed with a single question, “How committed are you to completing the exercise course?,” with answers given on a six-point Likert scale ranging from 0 = ‘no intention at all’ to 5 = ‘very strong intention.’ Intending to finish the program (score of at least 4) was a pre-defined inclusion criterion, thus reducing influences of the reflective system (i.e., intention) for adhering, while looking for differences in the impulsive system (i.e., AEE). Final analyses were based on data from 88 participants (24.98 years ± 6.88; 51.1% female).

#### Adherence Clusters

**FIGURE 1:**
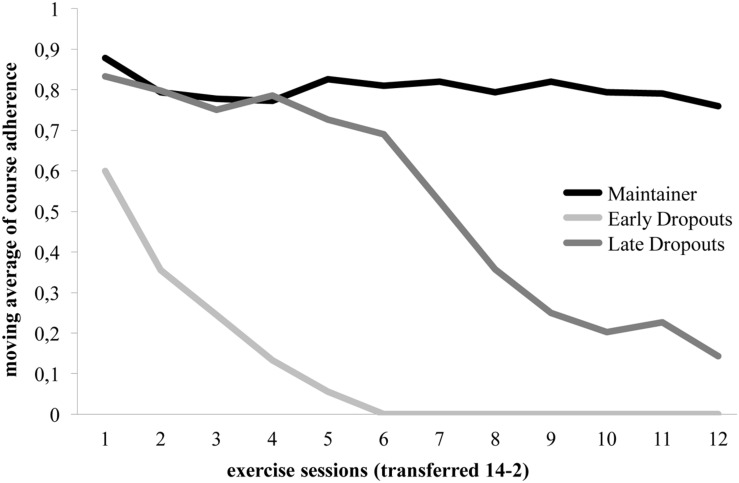
**Adherence groups with temporal development of course participation**.

In order to minimize bias in the data due to socially desirable responding (Kristiansen and Harding, 1984) and recall bias, attendance at the 14 exercise sessions was documented by the exercise instructor (present coded as ‘1’; absent coded as ‘0’) at the start of the session. Taking up the idea of Seelig and Fuchs (2011), we chose to refrain from the often used simple way of counting exercise sessions and calculating average participation rates and frequencies. Instead, we transformed our behavioral variable (whether a person is present or absent) to a categorical criterion measure and sought to identify typical adherence patterns. By doing so, we were able to depict behavioral qualities rather than artificial fractions of actually indivisible behavioral entities. Therefore the individual adherence data was transformed into 12 simple moving averages (each based on three sessions) per participant. The resulting series of moving averages were examined with hierarchical cluster analyses to identify different patterns of adherence in our group of participants. Three different adherence patterns emerged. Fifty-two participants were classified as ‘maintainers,’ 16 as ‘early dropouts’ and 20 as ‘late dropouts’ (giving an overall drop-out rate of 40.91% for the course). The results of this analysis and the chronology of adherence patterns are illustrated in **Figure 1**. Individuals of the maintainer group for example managed to at least reasonably adhere to the exercise program (78,85% attendance, range = 42.86–100.00%), whereas early dropouts stopped visiting the program during the first half of the 14-week exercise program (14.73% attendance, range = 7.14–35.71%) and late dropouts during the second half (56,01% attendance, range = 28.57–71.43%). The adherence groups did not differ with regard to age [*F*(2,82) = 1.28, *p* > 0.05]), gender [χ^2^(2) = 1.31, *p* > 0.05] or intention to participate in the course, *F*(2,85) < 1. Early dropouts (*M* = 175.33 min, *SD* = 112.75), late dropouts (*M* = 160.00 min, *SD* = 95.59) and maintainers (*M* = 202.75 mins, *SD* = 119.75) reported taking similar weekly volumes of exercise before the first course meeting [*F*(2,83) = 1.13, *p* > 0.05].

### Materials

#### Positive and Negative Associations

Automatic evaluations of exercising, positive and negative associations with exercise, were measured with a pictorial BIAT. The stimuli depicted scenes representing the target category ‘exercise’ (e.g., running, playing soccer, playing tennis, doing gymnastics) or non-sports activities (e.g., sleeping, watching TV, reading, sitting), i.e., the non-focal category. All stimuli were without obvious affective content (e.g., smiling faces). The evaluative categories ‘good’ and ‘bad’ were represented by eight (four and four) different smileys and frownies. Stimuli were presented in the middle of the screen and remained there until the participant categorized them by pressing the ‘E’ or ‘I’ key on a standard QWERTZ keyboard. In test block A (positive associative focus) participants had to press the ‘E’ key if the stimulus belonged to either the target category ‘exercise’ or the evaluative category ‘good’; they were asked to press the ‘I’ key in response to all other stimuli in this block. In test block B (negative associative focus) ‘exercise’ and ‘bad’ stimuli were assigned to the ‘E’ key and all others to the ‘I’ key. Block order was counterbalanced between participants. All participants started with 24 practice trials, followed by 40 trials with response time logging (Inquisit 2.0 software). They were instructed to categorize stimuli as quickly and accurately as possible. The resulting *D*-score was calculated with the revised scoring algorithm by Greenwald et al. (2003).

### Design and Procedure

Before the first exercise session potential participants were asked whether they were willing to take part in a study on ‘evaluations of exercising.’ Participants completed their first BIAT immediately before the start of the first exercise session. Then they completed a short paper–pencil questionnaire containing questions on intention of finishing the exercise course, weekly volume of exercise (in minutes per week) and basic socio-demographic information (age and gender). Finally, the course instructors documented the presence or absence of participants. Attendance was documented by instructors before each session throughout the 14 weeks of the exercise course. The participants attended a weekly exercise course that combined cardio training with exercises focusing on strength and coordination. Participants were fully debriefed, after the last exercise session. The study was carried out according to the recommendations of the ethical committee of the University of Potsdam.

### Statistical Analyses

Group differences in *D*-scores were assessed using one-way analysis of variance (ANOVA) and group differences in the separate positive and negative associative foci were analyzed with one-way multivariate analysis of variance (MANOVA) with the associative foci as dependent variables and the adherence groups as independent variables. Follow-up discrimination analysis (Field, 2013) was used as a *post hoc* test. This strategy allows to analyze the relative discriminative power for each of the two (inter)dependent variables ‘positive and negative associative foci’ with regard to the criterion variable adherence group (maintainer; early dropout; late dropout).

## Results

**FIGURE 2:**
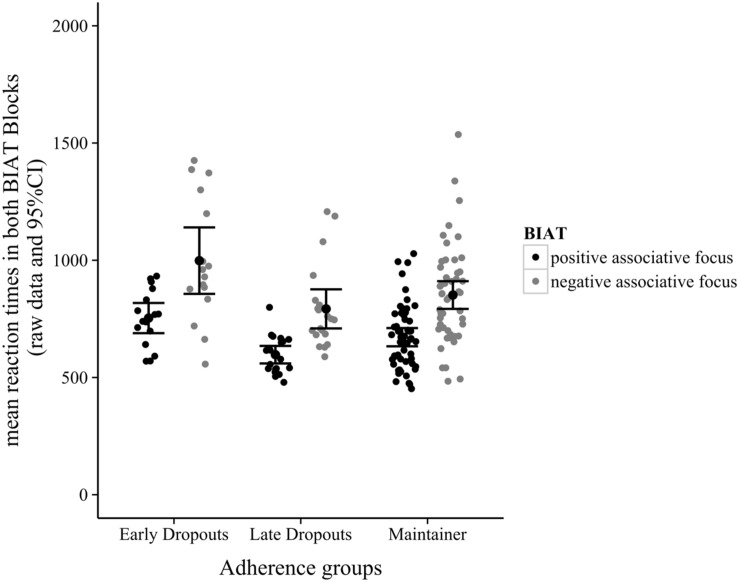
**Mean reaction times in both BIAT blocks**.

Full descriptive data are given in **Table 1**. ANOVA revealed that *D*-scores were similar for the three groups, *F*(2,85) = 0.57, *p* > 0.05, with early dropouts having the least positive AEE (*D*-score = 0.40). However, in the MANOVA there was an omnibus effect (Pillai’s trace) of adherence group on positive and negative exercise associations (**Figure 2**), *V* = 0.15, *F*(4,170) = 3.35, *p* < 0.01, $\eta_{p}^{2}$ = 0.07. A follow-up discriminant function analysis revealed two functions, explaining 99.6% (canonical *R*^2^ = 0.16) and 0.4% (canonical *R*^2^ < 0.01) of the variance, respectively. The combination of the two discriminant functions differentiated between the three groups, *L* = 0.85, χ^2^(4) = 13.33, *p* < 0.01. Removing the first function revealed that the second function did not contribute significantly to the effect, *L* = 0.99, χ^2^(1) = 0.52, *p* > 0.05. Inspection of correlations between the independent variables and the two discriminant functions revealed that positive exercise associations were strongly positively loaded on the first function (*r* = 0.97) and weakly to moderately negatively loaded on the second function (*r* = -0.21); negative exercise associations were strongly positively loaded on the second function (*r* = 0.84) and less strongly positively loaded on the first function (*r* = 0.55). These results suggest that the two associative foci are differently associated with the adherence groups.

**Table 1:** Means and standard deviations of the dependent variables for the three adherence groups.

Dependent variables	Early dropouts	Late dropouts	Maintainers
Positive associative focus	753.43	597.28	671.89
	(120.83)	(79.93)	(139.80)
Negative associative focus	998.37	792.67	905.11
	(266.10)	(178.13)	(339.95)
*D*-score	0.40	0.55	0.52
	(0.47)	(0.28)	(0.51)
Intention	4.88	4.85	4.77
	(0.34)	(0.37)	(0.40)

*Values in braces are the standard deviations of the adherence groups mean reaction times, *D*-scores or intention measure.*

## Discussion

The aim of this study was to examine individual differences in positive and negative associations with exercise in exercisers who started a 3-month program of weekly exercise sessions. Analysis of adherence to the program uncovered three groups of exercisers, maintainers, early dropouts and late dropouts. We detected no statistically significant differences on the level of average *D*-scores. However, the strengths of positive and negative associations with exercise measured before the start of the exercise program differed significantly and with a medium-sized effect between the three adherence groups. In order to better understand the differences between the two associative foci and the adherence groups, a discriminant function analysis was conducted. This approach is recommended, since MANOVA examines “whether groups differ along a linear combination of outcome variables and discriminant function analysis, unlike ANOVA, breaks down the linear combination in more detail” (Field, 2013, p. 654).

Discriminant function analysis revealed that a combination of the two underlying types of exercise association (positive and negative) was highly effective in discriminating between the three adherence groups. Positive exercise associations contributed more to adherence group classification. The three groups were identified empirically (we described whether our participants were present or not) after our measurement of AEE; we thus conclude that AEE in the positive focus (not in the negative focus) are helpful in predicting exercise course adherence. Having a closer look at the positive associative focus, on the descriptive level, early dropouts seem to have the longest reaction times. This goes in line with our expectations. However, maintainers did not display the shortest reaction times (i.e., the most favorable exercise associations). There are several explanations for this result. First, the reliabilities of IATs are part of a lively debate (e.g., Banse et al., 2001) leading at least some researchers to the conclusion that indirect measures like the IAT should “be used with caution” (Bluemke and Friese, 2008, p. 977). The slightly faster reaction times of the late dropouts, compared to the maintainers, could be due to measurement error. A second explanation lies in our exclusive inspection of the automatic level, the two associative foci, while neglecting probable (well-evidenced) influences from the reflective system. An initial positive association with exercising seems to facilitate later exercise adherence, according to our results. Nonetheless, planning and coping skills remain important and might help exercisers to overcome negative automatic evaluations. Future studies are needed to focus on probable interactions between the impulsive and reflective components influencing exercise behavior.

Previous studies investigated the association between AEE and exercise behavior over a short time period (Conroy et al., 2010). Our findings extend understanding of the relationship between AEE and behavior in several ways. We suggest that adherence to a program of exercise can be described as a series of situated decisions of the form ‘do I attend my aerobics class today or watch TV instead?’ Earlier research has shown that AEE correlated significantly with situated decisions about exercising (Brand and Schweizer, 2015). Our data corroborate the hypothesis that long-term adherence to a program of exercise, i.e., repeated decisions to engage in exercise, and positive associations with exercising (associations between mental representations of ‘exercise’ and the evaluative category ‘good’) at the beginning of the course are correlated. This result is compatible with previous accounts of AEE and their role in physical activity behavior (e.g., Hyde et al., 2012).

In the terms of learning theory each exercise class represents a learning experience which influences the weights of associations between affective representations and exercise representations accordingly. A pre-existing positive AEE might act as a buffer against the effects of future exercise classes which might trigger predominantly negative affect. Deutsch and Strack (2006) posited that in long-term memory the weight of associations between, for example the concepts ‘exercising’ and ‘good’ change only slowly. If the stored evaluation of exercising is that it is ‘enjoyable,’ i.e., there is a stored association between exercising and positive affect which is reflected in a general motivation to engage in exercise, then it is likely that even if the individual has recently had an unpleasant (negative) experience of exercising his or her overall motivation to exercise will remain high (i.e., he or she is likely to make situated decisions to exercise, rather than undertake an alternative activity). This view is consistent with other authors’ findings on the correlation between directly assessed hedonic responses to exercise and adherence to a program of exercise (Williams et al., 2008; Ekkekakis, 2009; Kwan and Bryan, 2010). Williams et al. (2008, p. 232) concluded that “a positive affective response may lead to greater participation in physical activity programs” on the basis of an assessment of affective responses to an exercise session and follow-up tracking of physical activity for 6 months. We propose that the correlation between positive affect and exercise behavior is not only a matter of reflective evaluative judgments based on rational deliberation (e.g., ‘no pain, no gain’) but also automatic evaluations (i.e., spontaneous affective responses or ‘gut feeling’; the output of the impulsive system). This implies that exercise intervention practitioners should attempt to facilitate immediate, positive affective responses to exercise for participants in order to reinforce exercise-positive associations which may influence both impulsive and reflective processing of exercise-related stimuli and choices.

Our findings also contribute to understanding of AEE measurement. We suggest that it is more appropriate to conceive AEE in terms of separate exercise-positive and exercise-negative associations rather than as an overall AEE, on a single linear continuum. It is noteworthy that it is the overall linear continuum model which provides the rationale for calculation of IAT *D*-scores. Co-existing positive and negative associations and learning experiences in everyday life (e.g., exercising makes me feel better but at the same time it is time-consuming) are the norm rather than the exception. Our behavior is guided by this complex interplay of reflective judgments and automatic associations; both factors should be assessed in more detail when assessing patterns of complex behavior such as exercise habit. Assessing positive and negative associative foci separately supports a more nuanced interpretation of individual differences evaluations based on impulsive system processes. The lack of significant differences between the *D*-scores of maintainers and early and late dropouts reinforces the case for considering positive and negative automatic evaluations separately, particularly as differences between the adherence groups were detected when positive and negative associative foci were examined separately. Furthermore our results suggest that the positive and negative associative focus contribute differentially to patterns of exercise adherence. Given that we investigated individuals who already had decided to visit this exercise course it is unsurprising that most of them had positive associations involving exercise and that these positive associations had a significant impact on behavior. One would expect our participants to display strong or salient exercise-positive associations acquired as a result of numerous previous positive experiences of exercising (all opportunities for associative learning). As Strack and Deutsch (2006, p. 167) put it: “Frequently co-occurring perceptual features, valence, and behavioral programs form associative clusters, which vary in their accessibility according to the recency and frequency of their activation.” Future research should investigate inactive individuals in order to clarify the observed differential impact of positive and negative associative in individuals without the intention to exercise.

## Conclusion

We also conclude that our approach to describe exercise behavior on the full categorical level (i.e., visit the program or not) was successful. Many studies effectively described physical activities in terms of volume (e.g., step counts or minutes per week) and the observation that exercise volume correlates with AEE (e.g., Eves et al., 2007; Conroy et al., 2010) is certainly useful. This type of quantitative information neglected, however, qualitative behavioral differences, e.g., how similar volumes of exercise actually summed up. In a 14-week exercise session, individuals could either participate in every second exercise session (and thus be classified as a maintainer) or stop attending the exercise course after having been there for the first seven sessions (and thus belong to the late-dropout group). Accounting for such chronological information was fruitful and should stimulate further research and developments regarding the design of targeted exercise interventions (e.g., Keele-Smith and Leon, 2003).

Although the results of this study contributed to our understanding of AEE and their relationship with exercise behavior there are limitations to our study that need to be addressed. The moderate sample size of 88 participants needs to be mentioned. Since our study was embedded in an actual 14-week exercise program with uncertain attendance, we abstained from calculating an *a priori* power analysis. However, a *post hoc* analysis of the achieved power (taking our given sample and effect size into account) resulted in a power of 0.86, what supports the significance of our results. The regular exercisers in our sample all reported that they were likely to attend the sessions regularly and it is important to be cautious about generalizing the findings to less motivated individuals. The relationship between AEE and adherence to an exercise program in less motivated individuals is a question for future empirical research. It is also unclear whether the same results would have been obtained when investigating the relationship between AEE and exercise for more than 14 weeks.

These limitations notwithstanding, we think that our study highlights the influence of AEE and the two underlying associations on adherence to a program of exercise. Our aim was to enrich understanding of the research issues in several ways. First, we have offered a plausible theoretical account of the relationship between situations-specific AEE and long-term adherence to an exercise program. This invites further reflections on integrating AEE into theories of exercise behavior. Dual-system models are one approach to doing so and provide a basis for future research into exercise habits. Second, we have provided evidence that AEE predict exercise behavior over the long term, thus extending previous findings which investigated exercise habits or exercise behavior over short time periods. Third, the decomposition of AEE into its components (i.e., exercise-positive and exercise-negative associations) was shown to be essential to understanding the relationship between exercise behavior and AEE. Our finding improves understanding of the concept of AEE and should lead to development of more effective exercise interventions. Mainstream research in exercise psychology should investigate automatic as well as reflective processes of behavior regulation in the future.

## Author Contributions

FA developed this research question. FA conducted the empirical part of the study. RB and FA jointly re-analyzed the data, adjusted and broadened the chain of arguments and then cooperatively wrote this report.

## Conflict of Interest Statement

The authors declare that the research was conducted in the absence of any commercial or financial relationships that could be construed as a potential conflict of interest.
